# Prediction of small-for-gestational age by fetal growth rate according to gestational age

**DOI:** 10.1371/journal.pone.0215737

**Published:** 2019-04-26

**Authors:** Min-A Kim, Gwan Hee Han, Young-Han Kim

**Affiliations:** 1 Department of Obstetrics and Gynecology, Gangnam Severance Hospital, Institute of Women’s Life Medical Science, Yonsei University College of Medicine, Seoul, Korea; 2 Department of Obstetrics and Gynecology, Severance Hospital, Institute of Women’s Life Medical Science, Yonsei University College of Medicine, Seoul, Korea; University of Liverpool, UNITED KINGDOM

## Abstract

**Background:**

Small-for-gestational age (SGA) infants should be identified before birth because of an increased risk of adverse perinatal outcomes. The objective of this study was to assess the impact of fetal growth rate by gestational age on the prediction of SGA and to identify the optimal time to initiate intensive fetal monitoring to detect SGA in low-risk women. We also sought to determine which the ultrasonographic parameters that contribute substantially to the birthweight determination.

**Methods:**

This was a retrospective study of 442 healthy pregnant women with singleton pregnancies. There were 328 adequate-for-gestational age (AGA) neonates and 114 SGA infants delivered between 37+0 and 41+6 weeks of gestation. We compared the biparietal diameters (BPD), head circumferences (HC), abdominal circumferences (AC), femur lengths (FL), and estimated fetal weights (EFW) obtained on each ultrasound to determine which of these parameters was the best indicator of SGA. We created receiver operating characteristic curves, calculated the areas under the curves (AUCs), and analyzed the data using multivariable logistic regressions to assess the ultrasound screening performances and identify the best predictive factor.

**Results:**

Among the four ultrasonographic parameters, the AC measurement between 24+0~28+6 weeks achieved a sensitivity of 79.5% and a specificity of 71.7%, with an AUC of 0.806 in the prediction of SGA. AC showed consistently higher AUCs above 0.8 with 64~80% sensitivities as gestational age progressed. EFW measurements from 33+0~35+6 gestational weeks achieved a sensitivity of 60.6% and a specificity of 87.6%, with an AUC of 0.826. In a conditional growth model developed from the linear mixed regression, the value differences between AC and EFW in the SGA and AGA groups became even more pronounced after 33+0~35+6 weeks.

**Conclusion:**

Healthy low-risk women with a low fetal AC after 24 weeks’ gestation need to be monitored carefully for fetal growth to identify SGA infants with a risk for adverse perinatal outcomes.

## Introduction

Small-for-gestational age (SGA) infants are at increased risk of adverse perinatal outcomes such as low Apgar scores, neonatal intensive care unit (NICU) admissions, neurological injuries, stillbirths, and neonatal deaths. Therefore, it is imperative to identify SGA fetuses prior to delivery. Assessing fetal size via ultrasound is the most commonly used and best method available in clinical obstetric practice. Ultrasonography also plays an important role in planning and guiding obstetric interventions for fetuses that exhibit abnormal growth rates [1]. SGA infants identified during the antenatal period that receive appropriate and timely interventions have a four-fold decrease in composite severe morbidity and mortality [2]. Therefore, detecting abnormal growth patterns that can lead to fetal growth restriction and SGA is extremely important during the prenatal period.

Despite a lack of evidence regarding the effects of gestational age on fetal weight, it is important to identify the correct gestational age to predict SGA on routine second and third trimester ultrasounds. Obstetricians increasingly perform ultrasound examinations to identify SGA at what is currently considered the optimal point in gestation. Although, SGA is strongly related to hypertensive disorders, half of all SGA infants are delivered by women considered to be low-risk. However, there have been debates about the effectiveness of birthweight screening in low-risk pregnant women, and the usefulness of detecting small fetuses [3–5]. Measuring the fundal height is traditionally used to assess fetal growth during outpatient prenatal care visits, although it is reported to have low sensitivity (10%) and a specificity of 96% [6]. However, an ultrasound scan is significantly better for detecting SGA than abdominal palpation. Nevertheless, the optimal timing of such a scan is uncertain. Recently, a few studies that included women with low-risk pregnancies have been conducted to determine the value of sonographic fetal biometry for predicting SGA during the mid-second and third trimesters. However, controversies still exist as to the proper reference ranges or standards for fetal size and growth, the timing for fetal biometry to estimate fetal weight, and the specific ultrasonographic parameters that play a role in predicting SGA.

The objective of this study was to find the optimal time to initiate intensive fetal weight monitoring and to determine the most predictive ultrasound parameter to identify SGA fetuses among low-risk pregnant women. This valuable knowledge could be used to provide specialized antenatal care and potentially decrease SGA-related perinatal mortality and morbidity.

## Materials and methods

This was a retrospective study in which data were collected from the medical records of 442 healthy pregnant women with singleton pregnancies who delivered livebirths between 37+0 and 41+6 weeks of gestation at the Gangnam Severance Hospital between January 2015 and March 2017. Each patient had routine mid-second and third trimester ultrasound examinations for fetal biometry, which is common practice. The 442 women in the study delivered 328 adequate-for-gestational age (AGA) neonates and 114 SGA neonates. The study was approved by the Institute of Review Boards (IRBs) of Gangnam Severance Hospital.

The patient characteristics that were recorded and analyzed included maternal age, maternal body mass index (BMI) before pregnancy and at delivery, maternal weight gain during pregnancy, parity, neonatal birthweight, gestational age at delivery and at each ultrasound measurement, and fetal biometrics with estimated fetal weights (EFW) in each divided period. The gestational ages were determined from the fetal crown-rump length measurements at 8–9 weeks of gestation. Serial ultrasound examinations were performed every four weeks (± one week) until delivery. Thus, the gestational ages used in the analysis were measured during the following three gestational age windows: 24+0~28+6, 29+0~32+6, and 33+0~35+6 weeks. The gestational age after 36 weeks was excluded from the analysis because the gestational age at the time of delivery was included. Serial ultrasound measurements included the following biometric parameters: fetal biparietal diameter (BPD), head circumference (HC), abdominal circumference (AC) and femur length (FL). We performed all ultrasound measurements based on the recommendations of the International Society of Ultrasound in Obstetrics and Gynecology. The EFW was calculated according to the Hadlock formula:

[log10EFW=1.335−0.0034×(AC)×(FL)+0.0316×(BPD)+0.0457×(AC)+0.1623×(FL)].

[7]

Depending on the gestational age at birth, the diagnosis of SGA was made if the birthweight was below the 10^th^ percentile for that gestational age, with reference to customized growth percentile charts developed by Hadlock et al [8]. AGA was defined as a birthweight between the 10^th^ and the 90^th^ percentile according to the same growth chart. We excluded pregnant women with any of the following antenatal complications: hypertensive disorders of pregnancy or preeclampsia, pre-existing diabetes mellitus or gestational diabetes mellitus, preterm delivery, twin pregnancy, chromosomal anomaly, structurally abnormal fetus, and early-onset growth restriction before the routine mid-second or third trimester ultrasound.

All data were tested for distribution normality using the Shapiro-Wilk test. The demographic characteristics were compared using the independent *t*-test for continuous variables and are presented as means ± standard deviations (SD). Categorical variables were compared using the chi-square test or Fisher’s exact test and are presented as numbers and percentages. We performed an analysis of covariance (ANCOVA) for the fetal biometric data with the gestational age at the time of measurement as the covariate. A linear mixed model for repeated measures was used to examine the impact of the study population on the fetal biometric parameters over the measurement time. A receiver operating characteristic (ROC) curve analysis was performed to calculate the specificity and sensitivity of the fetal biometric parameters in the prediction of SGA. The diagnostic performance was determined from area under the curve (AUC) statistics for the entire ROC curve. We determined the best cutoff value as the point that maximized the Youden index (defined as Y = sensitivity+specificity-1). Univariate and multivariate logistic regression analyses were conducted to identify the adjusted risk factors associated with SGA. Potential nonlinear associations between the fetal biometric parameters and the measurement periods were assessed using a conditional growth model from the linear mixed regression. For all analyses, a P-value < 0.05 was considered statistically significant. All statistical analyses were performed using SAS version 9.3 (ASA Institute Inc., Cary, NC, USA).

## Results

The study population consisted of 442 pregnant women with singleton gestations, who underwent serial ultrasonographic examinations from the 24^th^ gestational week until the time of their full-term deliveries in Gangnam Severance Hospital. We included 114 women who delivered SGA neonates (SGA group) and 328 who delivered AGA neonates (AGA group) after 37 weeks of gestation. The demographic characteristics of the study population are shown in Table 1. There were significantly lower BMIs before pregnancy and at delivery in the SGA group than those in the AGA group. Also, the SGA group had little overall maternal weight gain during pregnancy compared to the AGA group, and the difference was significant. There was a significant difference in birth weight (3309.9 g) in the AGA group compared to the SGA group (2260.1 g) (p<0.001). However, there was no significant difference in the gestational ages at delivery between the two groups.

**Table 1:** Demographic characteristics of the study population.

Variables	AGA (N = 328)	SGA (N = 114)	p-value
Maternal age (years)	33.2±3.8	33.2±3.8	0.928
Maternal weight before pregnancy (kg)	55.2±7.8	52.2±8.0	<0.001
Maternal height (cm)	162.0±4.4	161.0±4.8	0.04
BMI before pregnancy (kg/m^2^)	21.0±2.8	20.2±2.8	0.005
Maternal weight gain during pregnancy (kg)	11.4±4.6	10.7±4.0	0.135
Maternal weight at delivery (kg)	66.6±8.4	63.0±8.9	<0.001
BMI at delivery (kg/m^2^)	25.4±3.0	24.3±3.1	0.001
Gestational age at delivery (days)	275.0±7.1	276.3±7.5	0.123
Parity			0.088
Nulliparous	207 (63.1)	82(71.9)	
Multiparous	121 (36.9)	32(28.1)	
Birth weight (g)*	3309.9±13.7	2660.1±23.2	<0.001

AGA, adequate for gestational age; SGA, small for gestational age; BMI, body mass index

Values are presented as mean ± standard deviation or n (%) unless otherwise specified.

*Values are presented as mean ± standard error after adjustment for gestational age at delivery.

Table 2 shows the fetal biometric parameters for BPD, HC, AC, FL, and EFW for the study population according to the gestational age ranges. The means of all the ultrasonographic parameters were lower in the SGA group than those in the AGA group with significant differences. Moreover, the mean BPD, AC, FL, and EFW values derived using the linear mixed model showed significant differences between the SGA and AGA groups as the gestation progressed.

**Table 2:** Fetal biometric parameters of the study population according to the gestational age ranges.

Parameters	Gestational age (weeks)	AGA (N = 328)	SGA (N = 114)	p-value	p-value*
BPD (cm)	24+0–28+6	6.63 ± 0.018	6.4 ± 0.034	<0.001	0.0499
	29+0–32+6	7.78 ± 0.019	7.57 ± 0.034	<0.001	
	33+0–35+6	8.59 ± 0.019	8.31 ± 0.033	<0.001	
HC (cm)	24+0–28+6	24.29 ± 0.052	23.68 ± 0.097	<0.001	0.0778
	29+0–32+6	28.3 ± 0.06	27.81 ± 0.11	<0.001	
	33+0–35+6	30.93 ± 0.057	30.27 ± 0.098	<0.001	
AC (cm)	24+0–28+6	21.98 ± 0.071	20.96 ± 0.13	<0.001	<0.001
	29+0–32+6	26.48 ± 0.066	25.34 ± 0.12	<0.001	
	33+0–35+6	30.06 ± 0.070	28.72 ± 0.12	<0.001	
FL (cm)	24+0–28+6	4.67 ± 0.014	4.56 ± 0.026	<0.001	0.0029
	29+0–32+6	5.6 ± 0.016	5.46 ± 0.029	<0.001	
	33+0–35+6	6.31 ± 0.014	6.12 ± 0.025	<0.001	
EFW (g)	24+0–28+6	908.07 ± 5.68	819.87 ± 10.55	<0.001	<0.001
	29+0–32+6	1566.02 ± 9.94	1409.38 ± 17.77	<0.001	
	33+0–35+6	2281.39 ± 11.14	2021.58 ± 19.22	<0.001	

Values are presented as mean ± standard error after adjustment for gestational age at measurement.

* Based on linear mixed model analysis with the study population and measurement periods

AGA, adequate for gestational age; SGA, small for gestational age; BPD, biparietal diameter; HC, head circumference; AC, abdominal circumference; FL, femur length; EFW, estimated fetal weight

**Fig 1:**
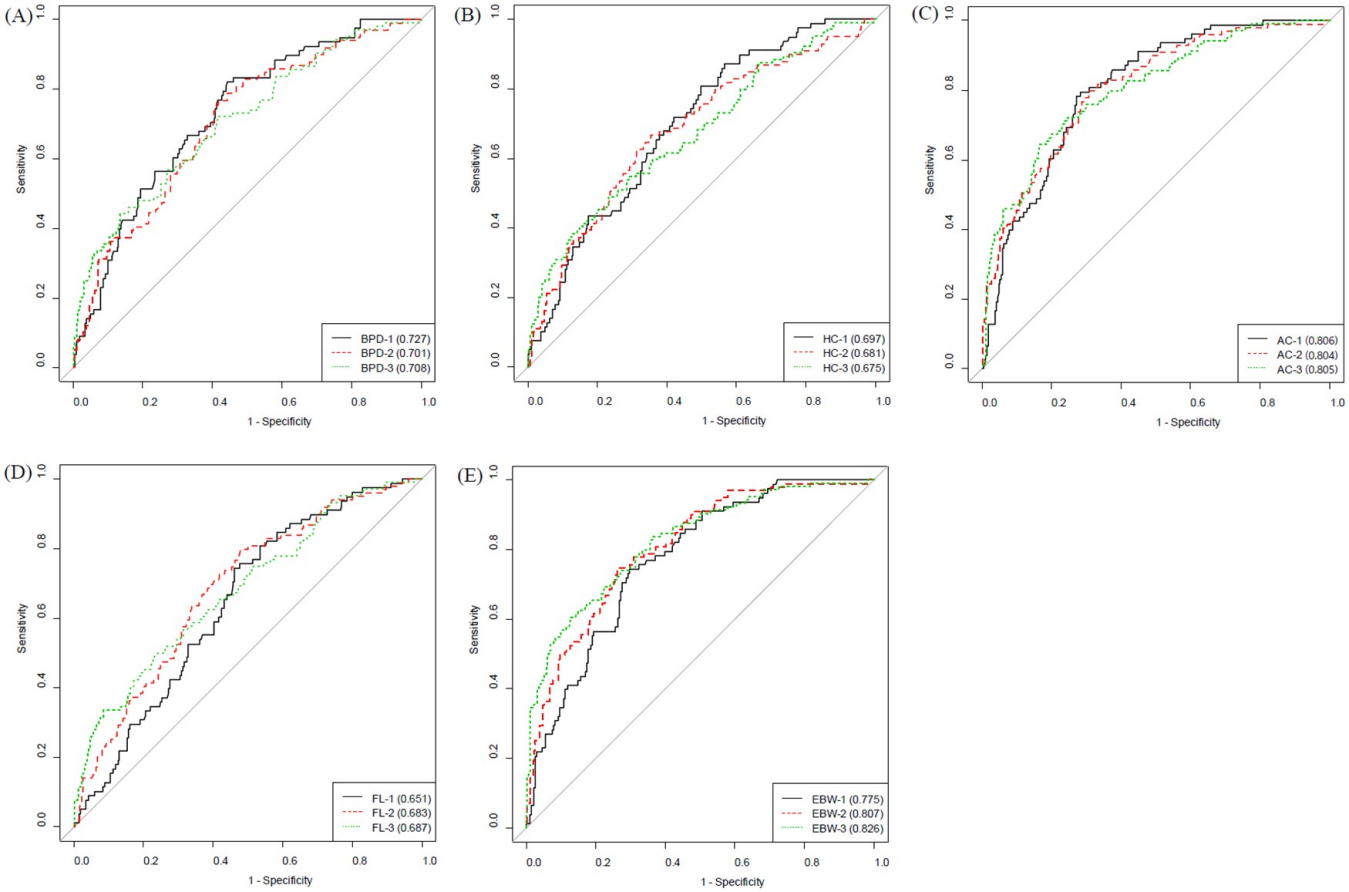
Receiver operating characteristic (ROC) curve of fetal biometric parameters predicting small for gestational age according to the gestational age ranges. **(A) BPD (B) HC (C) AC (D) FL (E) EFW.** The period 1, 2, 3 mean 24+0~28+6, 29+0~32+6, and 33+0~35+6 weeks of gestation, respectively. BPD, biparietal diameter; HC, head circumference; AC, abdominal circumference; FL, femur length; EBW, estimated fetal weight.

The diagnostic performance of the fetal biometric parameters in predicting SGA according to the gestational age ranges is shown in Fig 1 and Table 3. The EFW measured during the 33+0 ~ 35+6 gestational age range exhibited the highest AUC. The AUC was 0.826 with a sensitivity of 60.6% and a specificity of 87.6%. The positive predictive value (PPV) of the EFW was 61.2% and the negative predictive value (NPV) was 86.8%. The AC measured during the 24+0 ~ 28+9 gestational age range exhibited the next highest AUC. The AUC for AC was 0.806 with a sensitivity of 79.5% and a specificity of 71.7%. The PPV was 44.9% and the NPV was 92.3%. The AC measurements exhibited consistent AUC values above 0.8 over the three periods. Moreover, the AUCs for BPD and HC were the highest in the 24+0 ~ 28+6 gestational age range, and the AUC for the FL was the highest in the 33+0 ~ 35+6 gestational age range.

**Table 3:** Diagnostic performance of fetal biometric parameters for the prediction of small for gestational age according to the gestational age ranges.

	Gestational age (weeks)	AUC (95% CI)	Sensitivity (%)	Specificity (%)	PPV (%)	NPV (%)
**BPD**	24+0–28+6	0.727 (0.664–0.786)	82.1	55.8	35.0	91.5
	29+0–32+6	0.701 (0.64–0.764)	76.8	58.3	36.7	88.8
	33+0–35+6	0.708 (0.647–0.762)	44.2	86.4	52.3	82.2
**HC**	24+0–28+6	0.697 (0.631–0.753)	80.8	50.2	32.0	90.0
	29+0–32+6	0.681 (0.613–0.741)	62.6	68.8	38.8	85.4
	33+0–35+6	0.675 (0.61–0.732)	49.0	76.7	41.5	81.7
**AC**	24+0–28+6	0.806 (0.756–0.856)	79.5	71.7	44.9	92.3
	29+0–32+6	0.804 (0.75–0.851)	79.8	69.4	45.1	91.6
	33+0–35+6	0.805 (0.756–0.848)	64.4	83.5	56.8	87.5
**FL**	24+0–28+6	0.651 (0.585–0.712)	74.4	53.9	31.9	87.9
	29+0–32+6	0.683 (0.624–0.745)	79.8	52.2	34.5	89.1
	33+0–35+6	0.687 (0.63–0.743)	49.0	77.0	41.8	81.8
**EFW**	24+0–28+6	0.775 (0.718–0.828)	74.4	70.3	42.0	90.4
	29+0–32+6	0.807 (0.754–0.854)	74.7	73.9	47.4	90.3
	33+0–35+6	0.826 (0.778–0.872)	60.6	87.6	61.2	86.8

AUC, area under the curve; CI, confidence interval; PPV, positive predictive value; NPV, negative predictive value; BPD, biparietal diameter; HC, head circumference; AC, abdominal circumference; FL, femur length; EBW, estimated fetal weight

Table 4 the shows multivariable logistic regression analysis for the association between SGA and maternal characteristics, and the ultrasonographic parameter identifying determinants of SGA. The AC, which had the highest AUC, was significantly associated with SGA in all three gestational age periods, even after it was corrected by the pre-pregnancy and delivery BMIs (p <0.001).

**Table 4:** Adjusted risk factors for small for gestational age according to the gestational age ranges.

	OR (95% CI)	p-value	OR (95% CI)	p-value	OR (95% CI)	p-value
**BMI before pregnancy**	0.908 (0.809–1.018)	0.098	0.927 (0.844–1.017)	0.11	0.902 (0.818–0.995)	0.039
**AC**						
**24+0–28+6**	0.296 (0.202–0.435)	<0.001				
**29+0–32+6**			0.368 (0.279–0.485)	<0.001		
**33+0–35+6**					0.399 (0.314–0.506)	<0.001
**BMI at delivery**	0.91 (0.825–1.003)	0.057	0.903 (0.827–0.986)	0.023	0.9 (0.824–0.983)	0.019
**AC**						
**24+0–28+6**	0.297 (0.202–0.437)	<0.001				
**29+0–32+6**			0.368 (0.279–0.486)	<0.001		
**33+0–35+6**					0.397 (0.312–0.504)	<0.001

OR, odds ratio; CI, confidence interval; BMI, body mass index; AC, abdominal circumference

**Fig 2:**
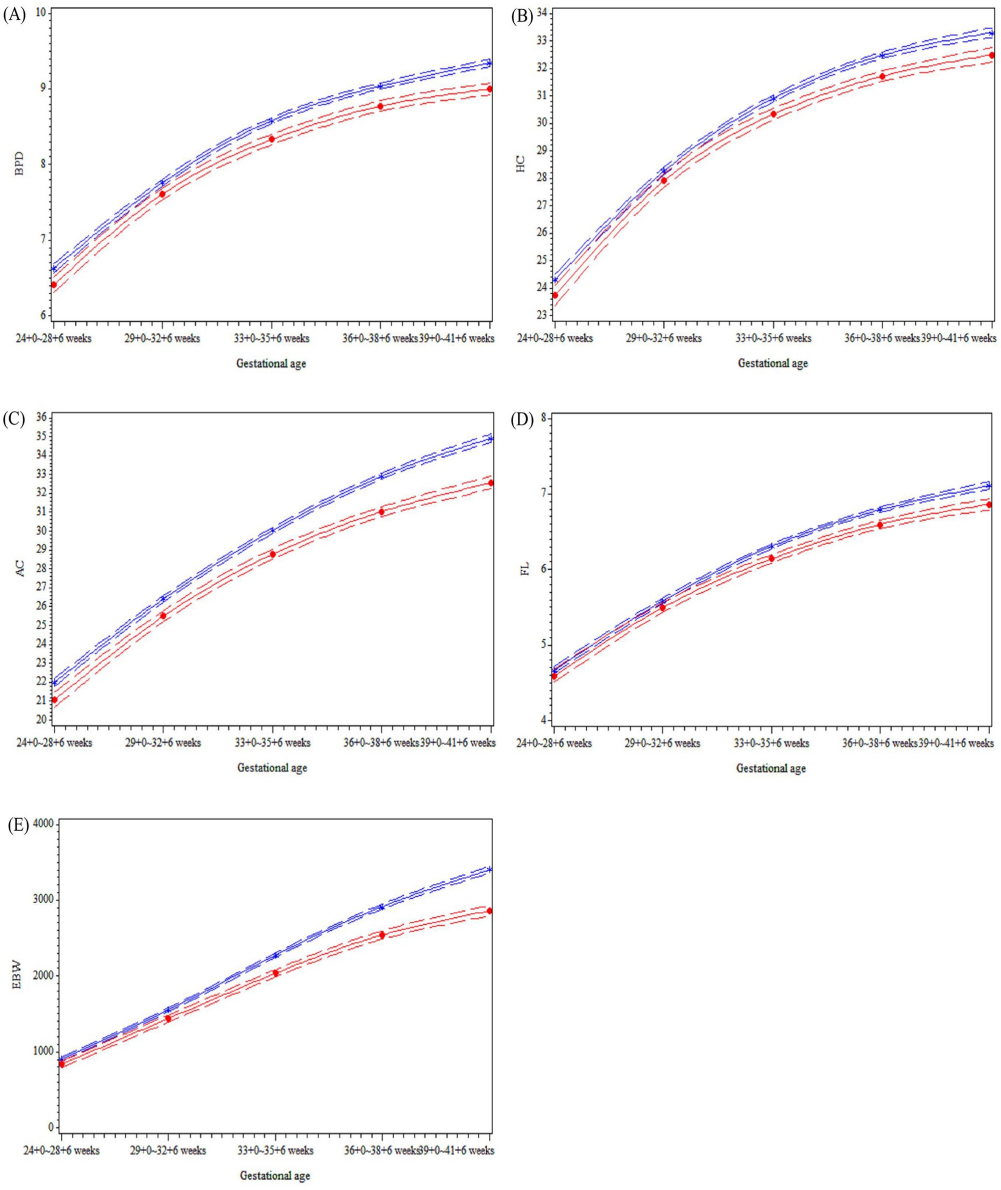
Distribution of ultrasonographic parameters and estimated fetal weight of the study population according to the gestational age ranges. **(A) BPD (B) HC (C) AC (D) FL (E) EFW.** AGA, adequate for gestational age; SGA, small for gestational age; BPD, biparietal diameter; HC, head circumference; AC, abdominal circumference; FL, femur length; EFW, estimated fetal weight.

We estimated fetal growth velocity for the AGA and SGA groups using a conditional growth model derived from the linear mixed regression during the five periods. Fig 2 shows that all the ultrasonographic parameters in both groups have a slightly convex shape, with the convexity appearing much less pronounced after 33+0 ~ 35+6 weeks. Specifically, the slope was less steep in the SGA group than that in the AGA group, and the difference in the AC and EFW between the SGA and AGA groups became even more pronounced after 33+0 ~ 35+6 weeks.

## Discussion

We evaluated the true efficacy of ultrasonography in the prediction of SGA. We investigated the optimal timing for the mid-second and third trimester ultrasounds and the primary biometric parameters facilitating the diagnosis and monitoring of SGA. The findings in this study demonstrated that intensive fetal growth rate assessments and monitoring to predict SGA infants are best conducted via ultrasound measurements of AC at 24+0 ~ 28+6 weeks of gestation. Unrecognized SGA fetuses had a four-fold higher risk of adverse outcome compared with those that were recognized antenatally [9]. Therefore, the antepartum recognition of SGA can improve fetal outcome. The period in which EFW is most predictive in the assessment and monitoring for SGA is 33+0 ~ 35+6 weeks of gestation. AC at 24+0 ~ 28+6 weeks of gestation had a higher sensitivity (80%) for the detection of SGA neonates than EFW at 33+0 ~ 35+6 weeks of gestation (61%), but the specificity was higher for EFW (88%) than AC (72%). Moreover, the AC measurement had higher AUCs (0.8) with 64~80% sensitivities as the gestation progressed. We also confirmed that the AC measurement was more closely correlated to birthweight and the detection of SGA than any of the other ultrasonographic parameters. All ultrasonographic parameters decreased after 24 weeks of gestation and the gap between SGA and AGA group was wider at 33+0 ~ 35+6 weeks’ gestation. On the basis of these results, if the fetal AC at 24+0~28+6weeks’ gestation is decreased in low-risk women without preeclampsia or other growth-affecting pregnancy conditions, subsequent intensive ultrasound monitoring could be helpful in the prediction of SGA neonates, thus decreasing the risk of SGA-related poor neonatal outcomes.

Currently, the performance of routine mid-second and third trimester ultrasounds for fetal biometry in low-risk pregnant women is controversial. Souka et al.[10] reported that in a low-risk group, an ultrasound for fetal biometry at 34–37 weeks was superior to an earlier third trimester ultrasound for the prediction of SGA (< 5th percentile) with 75% sensitivity for a 10% screen-positive rate. Fadigas et al. showed similar results, such that SGA prediction at 35–37 weeks of gestation by ultrasound examination was better than screening at 30–34 weeks with sensitivities of 70% and 58%, respectively. They also reported a false positive rate of 10% based on a combination of maternal characteristics and obstetrical history [11]. Roma et al. reported the usefulness of a routine third-trimester ultrasound examination for detecting fetal growth restriction as compared to ultrasounds performed at 32 and 36 weeks of gestation. They showed that the detection rate for fetal growth restriction was superior at 36 weeks compared to 32 weeks [12]. In contrast, the Cochrane database reported the effects of routine late pregnancy ultrasounds (defined as after 24 weeks’ gestation) on pregnancy outcomes in a review of eight trials including 27,024 women, which showed no significant improvements in perinatal mortality [3].

The present study showed that fetal AC was superior to BPD, HC, and FL in the prediction of SGA, which is in accordance with previous reports. Specifically, AC measured before 33 weeks had an almost 80% sensitivity in the detection of SGA. This was superior to EFW screening at 33+0 ~ 35+6 weeks, with a sensitivity of 64%. AC may therefore be clinically useful in the detection of SGA in the second trimester of pregnancy. Pronounced growth discrepancies after 33 weeks are additional indicators of an increased risk of SGA. The results of the present study are important because early identification of fetuses at increased risk for SGA could improve perinatal outcomes.

Tarca et al. have reported that single biometry measurements based on the last scan at < 32 weeks was 50% sensitive in the detection of SGA, which is almost identical to the sensitivity of serial measurements taken at least three weeks apart prior to 32 weeks [13]. However, performing multiple ultrasonography examinations to predict birthweight, as shown by Hedriana et al., was associated with a slight improvement in the prediction of birthweight compared with a single examination, particularly in the fetuses with abnormal growth [14, 15]. Some previous reports in high-risk populations defined by maternal characteristics, obstetric history, or EFW <10 percentile, proved that longitudinal assessments were associated with a reduction in adverse perinatal outcomes [16–18]. Recently, Sovio et al., in a large cohort study with 399 unselected nulliparous women, demonstrated a significant association between slow growth from the second to the third trimester and neonatal adverse outcomes [19]. Starting from 24+0 ~ 28+6 weeks, serial screening ultrasonography can help to detect the risk of growth deficits. Since the differences in EFW between the two groups were much greater after 33+0 weeks’ gestation, adverse perinatal events could be minimized with close ultrasonographic monitoring.

Among the ultrasonographic biometric parameters, AC was the best predictor for SGA, which is consistent with previous reports. Similar to our results, previous studies have shown that a slow AC growth rate is a risk factor for IUGR. De Reu et al, reported that the ultrasound examination at the beginning of the third trimester in a low-risk population could predict SGA (< 10th percentile) with 53% sensitivity and 81% specificity. They also reported that the most reliable cutoff for the prediction of SGA was an AC below the 25th percentile [20]. Skovron et al. reported that fetal ACs and EFWs obtained at 26–34 weeks’ gestation were equivalent predictors of SGA at birth compared with HC and the FL/AC ratio [21, 22]. Reboul et al. reported that a shorter ultrasound-to-delivery interval provided a better prediction of SGA than the third-trimester ultrasound scan. They also demonstrated that a single AC measurement was a better tool for predicting SGA than a single EFW measurement [23]. In our study, the AC measurement predicted the diagnosis of SGA based on the EFW by a mean of nine weeks. Therefore, once a small AC is detected, serial ultrasound monitoring for fetal growth in combination with maternal blood pressure measurements is useful for SGA monitoring.

The strength of this study lies in our discovery of the optimal timing to start intensive ultrasound monitoring to predict SGA. We found that the AC measurement is a strong predictor of SGA. There are limitations in our study. First, our study showed a higher prevalence of infants born SGA compared to other reports. It should be considered how the selection of the reference population would affect the prevalence of SGA. The Hadlock formula, commonly used in clinical practice in Korea, led to 25% of pregnancies being identified as having SGA in our study. In the past few decades, many studies have been conducted to develop references for fetal growth in different geographical areas [24–26]. There has been an ongoing debate over which reference should be adopted, but the formal verification is an essential process [27, 28]. Since the ethnic and regional characteristics of Western countries are fundamentally different from those of Asia, such application can cause the potential for misclassification. We should develop a national fetal growth standard in Korea for evaluating the features of high-risk neonate, which are different from those of Western countries. Second, the average birth weight in Korea has been declining since 1993. The average birth weight was 3,360g in 1993 and 3,190g in 2017, with a total decrease of 170g [29]. Recent in Korea, a decrease in the marriage rate, late marriage and childbearing and development of assisted reproductive technology are contributing to the increase in maternal ages [30]. Advanced maternal age has been associated with an increased risk of delivering a SGA neonate. Third, this effect may have resulted from the fact that the study was conducted at a tertiary center that women are referred to for specialist care by local clinics and that there were regional differences among patients with a high level of health awareness. Moreover, we did not analyze the association between the SGA group and neonatal morbidities that are actually related to the SGA group. The objective of the antenatal visit is to detect and minimize the risks of maternal and fetal morbidities and mortality. However, for SGA, sequential ultrasound screening remains controversial.

In conclusion, our study findings suggest that low-risk women with small fetal ACs after 24 weeks’ gestation should be monitored for AC growth as a predictor of SGA. These patients should also be offered an ultrasound for EFW after 33 weeks of gestation. To investigate this topic further, our results should be validated by large prospective studies and management protocols initiated when SGA is detected should be evaluated. Such protocols could reduce the high perinatal mortality and morbidity rates associated with SGA.
